# Reduction of acute respiratory infections in day-care by non-pharmaceutical interventions: a narrative review

**DOI:** 10.3389/fpubh.2024.1332078

**Published:** 2024-02-14

**Authors:** Lars Andrup, Karen A. Krogfelt, Lene Stephansen, Kristian Schultz Hansen, Brian Krogh Graversen, Peder Wolkoff, Anne Mette Madsen

**Affiliations:** ^1^The National Research Centre for the Working Environment, Copenhagen, Denmark; ^2^Department of Science and Environment, Molecular and Medical Biology, PandemiX Center, Roskilde University, Roskilde, Denmark; ^3^Gladsaxe Municipality, Social and Health Department, Gladsaxe, Denmark

**Keywords:** infectious transmission prevention, primary prevention, indoor air quality, aerosols, influenza, respiratory syncytial viruses, common cold, COVID-19

## Abstract

**Objective:**

Children who start in day-care have 2–4 times as many respiratory infections compared to children who are cared for at home, and day-care staff are among the employees with the highest absenteeism. The extensive new knowledge that has been generated in the COVID-19 era should be used in the prevention measures we prioritize. The purpose of this narrative review is to answer the questions: Which respiratory viruses are the most significant in day-care centers and similar indoor environments? What do we know about the transmission route of these viruses? What evidence is there for the effectiveness of different non-pharmaceutical prevention measures?

**Design:**

Literature searches with different terms related to respiratory infections in humans, mitigation strategies, viral transmission mechanisms, and with special focus on day-care, kindergarten or child nurseries, were conducted in PubMed database and Web of Science. Searches with each of the main viruses in combination with transmission, infectivity, and infectious spread were conducted separately supplemented through the references of articles that were retrieved.

**Results:**

Five viruses were found to be responsible for ≈95% of respiratory infections: rhinovirus, (RV), influenza virus (IV), respiratory syncytial virus (RSV), coronavirus (CoV), and adenovirus (AdV). Novel research, emerged during the COVID-19 pandemic, suggests that most respiratory viruses are primarily transmitted in an airborne manner carried by aerosols (microdroplets).

**Conclusion:**

Since airborne transmission is dominant for the most common respiratory viruses, the most important preventive measures consist of better indoor air quality that reduces viral concentrations and viability by appropriate ventilation strategies. Furthermore, control of the relative humidity and temperature, which ensures optimal respiratory functionality and, together with low resident density (or mask use) and increased time outdoors, can reduce the occurrence of respiratory infections.

## 1 Introduction

Infections account for around 50% of all sick leave, and acute respiratory infections are by far the most dominant cause (1–3). Adults experience on average between 2 and 4 annual infections of the respiratory tract and children between 4 and 8 (4) – especially children in day-care centers (DCCs) experience many infections in the first 6–12 months after they start (5). In Sweden about 95% of the episodes of children’s absence from DCCs are attributed to infectious diseases (6) and about 75% concerns various upper respiratory tract symptoms (7).

In DDCs in Denmark each child up to about 3 years has 23.7 sick days per year on average while children from about 3 years up to 6 years have 11 sick days per year on average (8).

A Scandinavian study found that 27% of days with infectious symptoms resulted in absenteeism from DDCs (9), so a large proportion of infected children continue to attend DCC. Overall, children attending DCC have more days of absence for sickness compared to children in family care, and this is most pronounced for younger children under the age of 3 years (10). Whether the total number of infections throughout life is higher when one has attended DCC is debatable (11).

In Denmark, DCCs are important to consider in relation to infection prevention, as around 86% of all children aged 1 year attend a public DCC or a state-supported private DCC (12). The total number of employees converted to full-time employees in public and private DDCs were in 2021 61,439 (13). Staff in DCCs, schools, and health sectors are frequently in contact with other people, and the massive exposure to infectious agents poses potential health implications for adult caretakers and parents of children attending DCCs. Research has shown that work in DCCs increases the risk of hospitalization due to pneumonia (14). Moreover, recent findings by Bonde et al. (15) reveal that childcare workers in Denmark faced an elevated risk of contracting COVID-19 during the pandemic, like that of healthcare workers. Notably, employees in DCCs and teachers exhibit one of the highest sickness absence rates in Denmark, with an average of 13.5 sick days per year compared to approximately 8 days per year for all employees (16). Thus, respiratory tract infections result in significant costs to society in the form of need for health care, productivity losses, poorer service and personal costs.

Various infection prevention measures may be used to reduce the spread of viruses and thus reduce illness and sickness leave. The COVID-19 era has given us new knowledge, not only about the corona virus and its variants, but also concerning transmission and prevention measures. This includes the virus’ ability of survival in the air and on surfaces, the excretion of small infectious droplets (aerosols) by various activities and the importance of temperature, humidity and targeted hygiene. This should be assessed for better prevention measures in DCCs, schools and other societal contexts with high occupant density.

The economic and health potential would be substantial if the recurrent respiratory infections can be reduced. This review addresses the following scientific questions: which respiratory viruses are the most significant in DCCs and similar indoor environments? What do we know about the transmission route of these viruses? What evidence is there for the effectiveness of different non-pharmaceutical prevention measures?

## 2 Methods

We performed a narrative review on respiratory infections and measures for mitigating transmission and disease with special focus on children and employees in day-care. Literature searches with different terms related to respiratory infections in humans, mitigation strategies, viral transmission mechanisms, and with special focus on day-care, kindergarten or child nurseries, were conducted using the PubMed database and Web of Science until august 2023. Searches with each of the main viruses (rhinovirus, respiratory syncytial virus, influenza virus, coronavirus, and adenovirus) in combination with transmission, infectivity, infectious spread were conducted separately supplemented through the references of articles that were retrieved.

Examples of the search criteria were:

- (viral OR virus) AND infections[tiab] AND respiratory[tiab] AND (children[tiab] OR infants[tiab]) AND prevalence[tiab] (969 results in Pubmed)
- (“Respiratory Tract Infections/epidemiology”[Mesh] OR “Respiratory Tract Infections/microbiology”[Mesh] OR “Respiratory Tract Infections/prevention and control”[Mesh] OR “Respiratory Tract Infections/transmission”[Mesh] OR “Respiratory Tract Infections/virology”[Mesh]) AND child daycare Centers[mesh] (376 results in Pubmed)
- ((RSV OR RS-virus) OR influenza virus OR coronavirus OR rhinovirus OR adenovirus) in combination with transmission, prevention or seasonality.
- Infections AND respiratory AND (daycare OR “day-care” OR kindergarten) (1,106 results in pubmed) in combination with one of the following terms: transmission, ventilation, “indoor air,” hygiene, “hand washing,” aerosols, humidity.

The search strategy was restricted to articles published in English or Danish. Potential articles were screened by title and abstract, and if relevant, the full text was assessed. Articles resulting from these criteria and relevant references cited in those articles were reviewed. All titles were screened by LA, and selected articles were read by LA and at least one other co-author. Inclusion criteria comprised relevant studies contributing to answering the formulated research questions and where the studies were assessed as being of acceptable quality and in peer reviewed journals. Both original studies and review articles were examined. Most emphasis was placed on systematic reviews, meta-reviews and scientific knowledge based on several sources. Where different studies show divergent scientific outcomes, a balanced description has been attempted *cf.* “best-evidence synthesis” approach (17) to minimize author’s bias. The article is structured according to the guidelines (Scale for the Assessment of Narrative Review Articles) for a narrative review described by Baethge et al. (18).

## 3 Results

The COVID-19 pandemic has shown the need to invest in infection prevention and the value of applying a range of strategies to reduce risk of transmission. In order to provide qualified strategies, knowledge of transmission routes, characterization of the most significant and abundant respiratory viruses, the impact of the indoor environment, and the seasonal patterns of the infection is of decisive importance.

### 3.1 Transmission route

The transmission of virus from an infected person to a recipient occurs primarily via the following pathways:

- Indirect transmission via physical contact with large aerosols (droplets) deposited on surfaces (fomites) and subsequent transfer to the recipient’s mucosae in the respiratory system primarily via hands or fomites.
- Airborne via large aerosols from the mouth of an infected person to the mouth, nose or eyes of the recipient.
- Inhalation of aerosols generated and released during breezing, speech, coughing and sneezing.

Emerging research, prompted by the COVID-19 pandemic, indicates that the primary mode of transmission for many respiratory viruses is through the air, in the form of tiny droplets known as aerosols or microdroplets (19). The term “aerosol” includes a diverse range of droplets and particles spanning a broad diameter spectrum, ranging from less than a nanometer to several hundred micrometers.

Human activities such as breathing, coughing, sneezing, or talking and singing result in the generation of aerosols by wind shear forces in the respiratory system. Variations in aerosol size arise due to differences in air pressure and velocity within distinct sections of the respiratory tract. The content of potential infectious virus depends, among other factors, on where the droplets originate in the respiratory tract.

In indoor environments, the transmission of infectious agents among a population, such as individuals in a DCC, is complex and may be influenced by several factors: I. The type of virus and its potential for airborne transmission (location and reproduction in the respiratory system and survival in aerosols); II. Concentration levels in respiratory system and aerosols; III. Size distribution of virus containing aerosol; IV. Characterization of the environment (temperature, humidity); V. Ventilation and air circulation pattern and VI. The number of virus shedding persons, their aerosol generating activities and exposure time.

The behavior of aerosols is primarily determined by the size of particles. Particles with diameters under 5 μm can linger in the air indefinitely under typical indoor conditions unless they are dispersed by air currents, ventilation, human movement, door openings, or body heat (20, 21). Aerosols with diameters <5 μm readily penetrate the airways down to the alveolar space, while particles with diameters <10 μm easily penetrate below the glottis. Large aerosols (droplets) with diameters greater than 20 μm exhibit a more ballistic trajectory, primarily governed by gravity, making them unable to follow the inhalation airflow streamlines due to their size. The Infectious Diseases Society of America (IDSA) suggests defining ‘respirable particles’ as those with a diameter of 10 μm or less and ‘inspirable particles’ as those with a diameter between 10 μm and 100 μm, with the majority being deposited in the upper airways (22).

In real-life scenarios, the relationship between aerosol size and infectivity forms a continuum, influenced by factors such as gravitational settling rate, transport, dispersion in turbulent air jets, viral load, viral shedding, and virus inactivation (23). Aerosols released into the air rapidly evaporate resulting in smaller particles. Depending on temperature, airflow, and humidity and the remaining salt and protein residue (dissolved substances) they can stay airborne for hours (24, 25).

Whether the infection is carried in small or large aerosols, the primary risk of aerosol transmission lies in proximity to infected individuals, as infectious virus particles in aerosols are diluted through ventilation and natural decay in the environment. This has been described as confusing and for many years has led to a lack of recognition that transmission through the air is the dominant route of transmission for respiratory viruses (26).

The airborne transmission of virus-containing saliva aerosols is a well-known mechanism for several respiratory viruses, including influenza (27), RS-virus (28, 29), rhinovirus (30) and *severe acute respiratory syndrome coronavirus 2* (SARS-CoV-2) (19, 31). A study showed that most particles (87%) with influenza virus RNA were smaller than 1 μm (32), see Table 1 for an overview.

**Table 1:** Principal respiratory viruses.^a^

	Rhinovirus	RS-virus	Influenza-virus	Coronavirus	Adenovirus
Size of virus	≈30 nm	120–300 nm	80–300 nm	≈120 nm	65–80 nm
Genome	RNA (7 kb)	RNA (15–16 kb)	RNA (segmented)	RNA (30 kb)	DNA (26–46 kb)
Baltimore classification^b^	IV	V	V	IV	I
Lipid envelope	No	Yes	Yes	Yes	No
Seasonality	All year but, peaks in autumn	Peak incidence from December to February^c^	Winter epidemic – peaks January to February^c^	Peak incidence from December to February^c^	Throughout the year. Peaks in late winter and spring
Transmission via aerosols	Yes (documented)	Yes (documented)	Yes (documented)	Yes (documented)	Yes (documented)
Transmission via hands	Likely in some situations (indicated^d^)	Likely	Likely (indicated^d^)	Likely in some situations (indicated^d^)	Likely
Transmission via fomite	Not likely (suggested)	Not likely (suggested)	Not likely	Not likely	Likely

^a^As identified in the survey of Nickbakhsh et al. (33).^b^Classification by route of genome expression (34).^c^In the northern hemisphere; yearly variations.^d^Limited evidence for this route in natural settings.

Different respiratory activities generate different sizes and numbers of aerosols. Breathing produces smaller particles than speaking and singing, suggesting that the use of the voice may carry a higher risk than simple breathing. The effects of the strength of the voice can be a factor of 20–30 increase in mass concentration of small aerosols (35).

### 3.2 Most important viruses

Several types of viruses can cause infections of the respiratory tract. Some are primarily infections of the upper airways, while others exhibit a greater predilection for the lower respiratory tract. Further, the transmission route, dose and airway functionality of the recipient, can influence the type and outcome of the infection. A further complication is that multiple viruses are often present in a respiratory disease course and asymptomatic persons are often carriers of the virus (36, 37).

In a large study from Scotland, analyzing 44,230 episodes of respiratory illness during 2005 to 2013, it was found, that five viruses were responsible for about 95% of respiratory infections: rhinovirus (RV), influenza virus (IV), respiratory syncytial virus (RSV), adenovirus (AdV) and coronavirus (CoV) (33). In a prospective cohort study of 119 children for 115 child years (mean age 10 months) attending day-care in the United States the most significant viruses were RSV, RV and AdV. These three viruses accounted for 67% of the viral infections (37).

#### 3.2.1 Rhinovirus (RV)

RVs are the leading cause of the common cold and significant contributors to exacerbations in chronic obstructive pulmonary disease (COPD) and asthma. They prompt more annual consultations than any other viral or bacterial respiratory source (4, 38–40). The manifestations of RV infections encompass mild upper respiratory tract illnesses (URTI) to more severe lower respiratory tract illnesses (LRTI). RV infections can additionally play a role in the development of otitis media and sinusitis (41). Repeated occurrences of RV infections in the early years of life may increase the risk of chronic respiratory conditions, such as asthma, later on (42).

RVs belong to the *Enterovirus* genus of the *Picornaviridae* family. There are more than 160 known genotypes of RVs (43). The virus is small (approximately 30 nm) and lacks a lipid envelope. The capsid proteins of the virus exhibit a high degree of heterogeneity, leading to a wide range of antigenic diversity (44).

RVs circulate consistently throughout the year, frequently reaching peak prevalence in the autumn and spring seasons. A prospective study of children attending DDC in the United States found no seasonal pattern of RV detection among children with respiratory tract infections (37).

In a study, it was observed that RVs were the predominant viruses in asymptomatic adults, constituting approximately 50%, with a notable prevalence during the summer months (45). Likewise, in children, it was observed that RV was the most prevalent virus, accounting for 71% of cases (46). A notable observation in Brazilian children was that, amid the COVID-19 lockdown and social distancing measures, the prevalence of the majority of respiratory viral pathogens was exceptionally low. However, RV persisted as the primary virus co-circulating with SARS-CoV-2 (47).

Given that the primary mode of RV transmission occurs indoors through the air, preventive strategies can center on enhancing ventilation, managing occupant density, and implementing measures to minimize aerosol generation and concentration while maintaining healthy airways. It is probable that, in certain environments, the contamination of hands might also play a role in the transmission of RVs (30). RVs are non-enveloped viruses, and ethanol sanitizers are less virucidal compared to organic acids (48).

#### 3.2.2 Respiratory syncytial virus (RSV)

RSV is the primary singular factor contributing to respiratory hospitalization in infants and stands as the second leading cause of mortality from lower respiratory infections worldwide (49, 50). RSV is very contagious, with nearly all children exhibiting signs of infection by the age of 2 (51). RSV infections lead to hospitalization in 0.5 to 1.0% of infected infants (52) and may be associated with the onset of wheezing and asthma in small children (53, 54). In addition, RSV is a frequently encountered cause of acute respiratory tract infections in adults (29) and resulting in a substantial economic burden on healthcare systems, governments, and society (55, 56).

RSV is an enveloped virus of medium size (120–300 nm) with a negative-sense, single-stranded RNA genome (15–16 kb). It belongs to the *Pneumoviridae* family and the *Orthopneumovirus* genus. There are two major antigenic groups, A and B (57, 58). Natural infection with RSV does not confer enduring immunity; as a result, reinfections are prevalent throughout an individual’s life. (59).

The incubation period for RSV infection is 2 to 8 days. RSV may remain confined to the upper respiratory tract, leading to symptoms like cough and runny nose, but more than 50% of initial infections in infants may progress to affect the lower respiratory tract several days later (60).

RSV circulates normally during the winter season, with its highest incidence typically occurring between December and January. A prospective study of children attending DDCs in the United States found RSV most often detected during fall and winter among children with respiratory tract infections (37). Typically, one of the two genotypes (A and B) tends to dominate in a given season, with an annual alternation or co-circulation (61). In some countries at northern latitudes, biennial variation with alternating severe and mild winter peaks have been observed (62). Therefore, a child born during a high-burden RSV season faces a hospitalization hazard 1.68 times higher than that of a child born in a low season (63).

The conventional understanding is that RSV transmission occurs through large aerosols (droplets) from infected individuals, entering mucous membranes of the eyes or nose through close contact or self-inoculation via touching contaminated surfaces (64). However, in recent years the spread of infection by inhalation of aerosolized airborne particles containing RSV has been increasingly acknowledged as a significant route of transmission, and the conclusion of the often-cited study from 1983 (64) that RSV transmission is more efficient at close range, favors both direct transmission and aerosol transfer (65). Studies have revealed that a substantial quantity of aerosolized particles containing infectious RSV can be detected in the vicinity of infants infected with RSV (28, 66), indicating the potential for aerosol transmission of the virus.

Amid the COVID-19 pandemic, a significant reduction in RSV infections (up to 70–90%) has been observed globally, likely attributed to containment measures like lock-down, face masks, hand hygiene, and social distancing. Primary prevention, limiting exposure to infectious agents, emerges as the most effective strategy in curbing the contagion and spread of SARS-CoV-2. It’s worth noting that handwashing agents containing detergents or alcohol are highly effective in eliminating RSV, while chlorhexidine without alcohol is not effective (67). Interestingly, it was found during the COVID-19 lockdown, that re-opening of schools was the predominant risk factor for RSV rebound. In addition, high temperature was demonstrated to decrease the risk for RSV (every 5°C increase reducing the risk by 37%) (68).

#### 3.2.3 Influenza virus (IV)

The 1918 influenza (*Spanish Flu*) killed 50–80 million people worldwide during three major waves (69). Since then, we have had three major influenza pandemics caused by emerged subtypes (Table 2). Influenza manifests as an acute respiratory illness marked by the abrupt onset of high fever, cough, headache, malaise, and upper respiratory tract inflammation. Individuals of all age groups are affected by influenza, with the highest occurrence seen in children. The most severe disease manifestations are observed in children, older adults, and individuals with preexisting health conditions.

**Table 2:** Influenza pandemics.

Pandemic	Years	Influenza type	Mortality
Spanish Flu	1918–1920	H1N1	50–80 million deaths (69)
Asian flu	1957–1959	H2N2	0.7–1.5 million deaths (70)
Hong Kong flu	1968–1969	H3N2	0.5–2 million deaths (71)
Swine flu (H1N1pdm09)	2009	H1N1	0.1–0.2 million deaths (72)

Epidemics lead to localized increases in infection rates, while pandemics are epidemics that extend globally. The persistent burden of seasonal influenza is substantial. A recent report estimates that 3–11% of individuals in the United States suffer from influenza each year (73). Additionally, influenza may contribute to 10–12% of total work-related sick leave (74, 75).

The Influenza virus is a member of the *Orthomyxoviridae* family, and within Influenza A viruses, subdivisions are based on antigenic characterization of the hemagglutinin (HA) and neuraminidase (NA) surface glycoproteins protruding from the virion. Sixteen HA and 9 NA subtypes are known (76). Two genetically and antigenically distinct lineages of influenza B viruses are found in humans. Influenza viruses have lipid-envelopes and contain eight RNA segments. Antigenic shift, facilitated by the segmented influenza virus genome, leads to a sudden and complete alteration of RNA-segments and HA and/or NA genes. This phenomenon, exclusive to influenza A viruses, is enabled by coinfection in animal reservoirs (aquatic birds and swine), allowing gene segment exchange between different subtypes. Antigenic shift can give rise to a virus for which the population lacks sufficient immunity.

Viral shedding of influenza in asymptomatic individuals has been observed in a comprehensive study conducted in Hong Kong. The proportion of asymptomatic cases ranged from 6 to 20%, depending on the influenza A virus subtype involved (77). In a systematic review, the combined estimate of the asymptomatic fraction among cases confirmed through virology was 16% (78). This implies that the influenza virus can be transmitted from infected individuals to their close contacts, even when there are no apparent clinical symptoms.

The relative importance of the different modes of transmission remains uncertain. However, as early as in 1941 it was demonstrated, that influenza virus could spread between cages in a ferret model, and Andrewes and Glover conclude: “Infection occurs over a distance of over 5 ft. in almost still air; it can even travel upwards and infect a normal ferret in a cage several feet above an infected animal. Good ventilation seems to interfere with the chances of infection” (79). There has been an increasing body of evidence supporting the potential for small aerosol transmission in recent years (80–82). Several studies have demonstrated the release of influenza RNA in the exhaled breath of individuals naturally infected with influenza (32, 83, 84). An example of airborne transmission of influenza virus (H3N2) was described in 1979, when an airplane with 54 people on board was delayed on the ground for 3 hours with inoperative ventilation. Within 72 h, 70% of the passengers became ill (85). Therefore, the most probable transmission mechanism is that both contact through hands and airborne routes are feasible, with the importance of each route varying in different situations.

Based on the correlation between temperature and relative humidity and transmissibility, Lowen and Palese hypothesized that transmission of influenza virus occurs through aerosols during winter season in temperate climates, but by direct contact transmission in tropical regions (86).

Protective measures, such as maintaining hand hygiene and using tissues for coughing and sneezing, are commonly advised during influenza epidemics and pandemics, although there is limited evidence supporting their effectiveness (87). The survival of the virus on human hands appears to be a critical factor in fomite transmission and numerous studies affirm that influenza viruses are rapidly inactivated on human hands. (88, 89).

#### 3.2.4 Coronavirus (CoV)

*Coronaviruses* are widely present viruses known to infect both humans and animals. They were initially identified in humans through research on the common cold in the 1960s. Before the current pandemic with the variant: *severe acute respiratory syndrome coronavirus-2* (SARS-CoV-2), human coronaviruses, such as HCoV-229E, HCoV-OC43, HCoV-NL63, and HCoV-HKU1 were well known. These *non-severe* strains cause seasonal infections with symptoms like “common cold.” Like other respiratory viruses, CoVs have been associated with otitis media (for a review see (90)). Coronaviruses are estimated to cause 15% of adult common colds. In temperate climates, HCoV-OC43 and HCoV-229E are predominantly observed in winter and have been associated with exacerbations of asthma and COPD in both children and adults (48).

Exposure is widespread during early childhood, and it is estimated that 90% of adults are seropositive for one or more CoV species (91). A study in Norway demonstrated that CoVs occurred in 1 of 10 hospitalized children with respiratory tract illnesses (RTIs) and the authors conclude that CoVs are associated with a substantial burden of RTIs in need of hospitalization (92).

In strong contrast to the non-severe strains, *severe acute respiratory syndrome coronavirus-1* (SARS-CoV-1), *Middle East respiratory syndrome coronavirus* (MERS-CoV) and SARS-CoV-2, which have emerged over the past 20 years, are more pathogenic (see Table 3 for overview).

**Table 3:** Major human corona strains.

Virus strain (genus)	Origin (year)	Route of transmission	Estimated mortality
SARS-CoV-2 (β)	China (2019)	Primarily via aerosol	< 4%
MERS-CoV (β)	Middle East (2012)	No strong evidence	34–36%
SARS-CoV-1 (β)	China (2003)	No strong evidence	9–10%
HCoV-HKU1 (β)	Seasonal circulation (2004/5)	No strong evidence	Probably very low
HCoV-NL63 (α)	Seasonal circulation (2004/5)	No strong evidence	Probably very low
HCoV-OC43 (β)	Seasonal circulation	No strong evidence	Probably very low
HCoV-229E (α)	Seasonal circulation	No strong evidence	Probably very low

^α^Belonging to the genus Alphacoronavirus.^β^Belonging to the genus Betacoronavirus.

An epidemic of SARS-CoV-1 occurred in spring of 2003 in East and Southeast Asia. Ultimately the pandemic spread to more than 20 countries and caused approximately 8,100 cases with 774 deaths (93). It seemed that young children experienced a less severe version of the illness (94).

The first case of MERS-CoV was identified in a patient in Jeddah, Saudi Arabia, in June 2012 (95). By 2019, 2,500 cases had been documented in 27 countries, resulting in 858 deaths with an estimated mortality rate of about 34% (96).

The still ongoing pandemic with SARS-CoV-2 causes major economic and health challenges in many countries worldwide.

The coronaviruses are enveloped, RNA viruses with a large genome (93). Human CoVs are zoonotic pathogens found in many animal species and may or may not cause disease symptoms in their hosts. The CoVs are divided into 4 genera of which the genera Alpha- and Betacoronaviruses mainly infect mammals (97). Coronaviruses undergo frequent genetic recombination, and if animals harbor different strains, this can lead to the evolution of new variants. It appears that such events have created SARS-CoV-2 (97).

It is assumed that transmission of the non-severe strains occurs via inhalation of aerosols or hands and fomite contact. Children can shed CoVs for longer periods after infection; however, data are limited. CoV infection due to the non-severe variants may occur year round with the highest incidences in winter and spring in temperate climates (48, 98).

In a large study, from the United States covering years 2014–2021, Shah et al. found that season onsets occurred October–November with peaks in January–February for the non-severe human coronaviruses. Most CoV detection (>93%) was within the defined seasonal onsets and offsets (99). Likewise, a study of hospitalized children in Norway showed that all CoV subtypes were primarily detected in winter, from November through March (92).

In 2003 a large outbreak of SARS-CoV-1 took place in a residential building complex with 19 buildings in Hong Kong. In total 331 cases were registered, and it was concluded that: “…airborne spread was the most likely explanation…,” and the virus may have spread over 200 meters. The index case apparently “shed” the virus through feces that were suspended in air, or aerosolized, by hydraulic action resulting in spread via the piping system (100, 101).

Overall, an increasing amount of knowledge documents that the most important route of transmission for SARS-CoV-2 is via the air in the form of large or small aerosols (82). Only relatively few people can infect many people with SARS-CoV-2 and only under the right circumstances (102). These individuals shed large amounts of infectious virus for a presumably short period during their illness. This has been seen in the so-called superspreader events, where a single person has infected numerous others in a few hours. These incidents have all been indoors and the infection seem to be spread via aerosols (103). For example, at one choir practice an individual infected 53 of 61 participants in a few hours (104). In another case, a person, dressed as a legendary figure for Christmas, infected 127 people at a nursing home in Belgium, and the authors conclude that airborne transmission was the most plausible explanation (105).

A recent systematic review found a lack of evidence demonstrating the recovery of viable virus from contaminated surfaces (fomites) suggesting that the risk of transmission of SARS-CoV-2 through fomites is low (106). Further, Weber and Stilianakis conclude: “…that virus inactivation on human hands could be a significant bottleneck, limiting fomite transmission risk of enveloped respiratory viruses” (107).

#### 3.2.5 Human adenoviruses (AdVs)

*Human adenoviruses* have the potential to induce a variety of symptomatic and asymptomatic infections that impact the respiratory tract, eyes, and gastrointestinal tract. During acute illness, adenoviruses may be excreted in substantial quantities in various bodily fluids, such as feces, oral secretions, and respiratory tract secretions. Globally, 5–10% of respiratory tract infections in children are ascribed to AdV (108, 109). Adenovirus infections frequently manifest in children aged between 6 months and 5 years; they are seen as febrile infections in the upper respiratory tract (110), for a review see (111).

One of the most common types (type 4) has been described to cause acute febrile illness, cough, hoarseness, sore throat, and constitutional symptoms (112). Children attending DCCs have been found to be a year-round reservoir of AdV and other viruses, and AdV was shed by 6% of the children (0–6 years). Interestingly, the likelihood of AdV shedding was tenfold higher among children who had received antibiotic treatment in the preceding 2 months (113).

Adenoviruses are members of the genus *Mastadenovirus* in the *Adenoviridae* family. Adenoviruses are non-enveloped and range in size from 65 to 80 nm in diameter (114). Today, about 100 AdV types that infect humans have been described (115), and grouped into seven species (AdV-A to -G). The tissue-specificity of the virus determines the manifestations of the infection. Various species have the capability to infect the conjunctiva, upper and lower respiratory tracts, and the gastrointestinal tract (108). Adenoviruses mostly cause self-limiting respiratory, gastrointestinal (GI), or conjunctival disease. Adenoviral respiratory infections are seen all year round, but have a highest incidence in late winter, spring and early summer (116).

The evidence for which transmission routes are the most significant under different conditions is scarce and presumably different for different species and mutants. A study of conjunctivitis with a strain of adenovirus demonstrated that the spread of infection was discontinued following infection control comprising, *inter alia* use of a surface disinfectant (0.5% sodium hypochlorite) that inactivates AdV (117). Inhaling small doses of AdV in aerosols led to the development of acute febrile respiratory illness, occasionally accompanied by pneumonia (118), consequently it was suggested, that purification of air in barracks rooms and other places with high occupant density, should diminish the spread of these infections (112). In an outbreak caused by AdV in a Military Hospital in Texas, it was concluded that droplets (large aerosols) were involved in the transmission of the virus (119). The virus can persist on environmental surfaces for prolonged durations and exhibits resistance to numerous disinfectants; nonetheless, it is deactivated by heat, formaldehyde, 95% ethanol, and bleach (111, 120, 121). The risk of respiratory pathogen infections is elevated by factors like the absence of preexisting immunity and crowding conditions. Environments where these factors exist include staff and children in DCCs (115).

### 3.3 Preventive measures relevant in day-care institutions

The extensive new knowledge that has been generated in the corona era has the potential to help prioritize the most cost-effective prevention measures in, for example, DDCs, schools and other societal contexts with high occupant density. Especially knowledge about the indoor air as a key transmission route may lead to a paradigm shift in the prevention of the spread for corona and the other respiratory viruses. Here, we present an overview of the evidence for the various preventive measures (Table 4).

**Table 4:** Non-pharmaceutical infection-reducing preventive measures in day-care centers.

Preventive measures related to building	Ventilation (mechanic/passive)	Effect against air borne transmission*: Yes
	Air filtration / disinfection	Yes
	Temperature / humidity	Yes
Preventive measures related to organization	Physical distance	Yes
	Occupant density	Yes
	Policy for sick leave, quarantine and work from home (social distance)	Yes
Preventive measures related to behavior	Hygiene practices (personal, building and objects) Mask wearing	Limited
		Yes
	Aerosol-generating activities (singing/talking, coughing, toilet flushing etc.)	Yes
	Physical activities	Yes
	Inside / outside time	Yes

* for references see text.

#### 3.3.1 Preventive measures related to building

##### 3.3.1.1 Ventilation

Airborne transmission of virus-containing aerosols is the dominant route of respiratory infectious diseases, such as COVID-19, influenza, and RV (19, 30, 122). Obviously, indoor air quality is essential for the prevention of potential transmission of disease. Ventilation can dilute the aerosol concentration in the air; filtration and, e.g., UV-C disinfection can add to the removal of potential pathogenic virus, and environmental factors like humidity and temperature can influence the survival of the virus in the air and modify the susceptibility of the recipient and the generation of exhaled aerosols/droplets from infectious persons (123, 124).

The infectious dose (number of viable virus particles required to induce an infection) depends on the specific virus variant and have been shown in some cases to be influenced by aerosol size (19). The goal is to reduce the concentration of aerosols containing infectious virus as much as feasible. Hence, enhancing the ventilation in a room or building offers a means to decrease exposure. Precautions should be taken, however, especially with the supply of dry cold air like during the winter season or from a heating and air conditioning system (HVAC), which may compromise the airways’ functionality, see below.

Ventilation involves the exchange of indoor air with fresh outdoor air. In certain situations, ventilation is employed to regulate the indoor thermal conditions, which includes temperature and air humidity, through the addition or removal of moisture, as well as by providing heating or cooling. Room ventilation can be natural or mechanical, often in combination with heating and air conditioning system configuration. The efficiency is measured in air changes/h (ACH). In addition, the ventilation can be supplemented with various types of filtrations (e.g., high efficiency particulate air (HEPA) cleaning units). The ACH can be in the range of less than 2 to over 6 in a well-ventilated room. A simulation study showed a 5% reduction of exposure per unit increase in ACH and that the addition of HEPA air cleaners significantly decreased exposure to aerosols (125).

We identified only two studies that compared ventilation effectiveness with prevention of respiratory infections in day-care settings. In a Danish study, Kolarik et al. (126) found that the total number of respiratory infections decreases by 12% every time the air exchange was increased by one ACH. In a study from Finland, otitis media was significantly reduced for young children in DCCs with mechanical ventilation compared to DCCs with only natural ventilation (127). However, there are methodological bias in both studies. In the Danish study, the children’s sickness absence was not measured in the same year as the ventilation efficiency, and in the Finnish study, the ventilation quality was only assessed qualitatively and not measured.

We identified several studies that have focused on schools and college residence halls, which in some respects can be compared to DDCs (128, 129). In a real-life situation, the risk of COVID-19 infections was significantly reduced among students in United States by improved ventilation. Schools that enhanced their ventilation systems experienced a 39% reduction in COVID-19 incidence, in contrast to schools that did not implement these preventive measures. Ventilation strategies comprised methods to dilute exposures by opening windows, opening doors, or using fans, or in combination with filtration methods with or without ultraviolet germicidal irradiation (48% lower incidence) (128).

In a recent extensive intervention-cohort study conducted in Italy, it was observed that classrooms equipped with mechanical ventilation systems experienced a relative risk reduction of at least 74% in student infections with SARS-CoV-2 compared to classrooms relying solely on natural ventilation (130).

Similarly in office buildings in the United States an apparent reduction of 35% in sick leave rates was associated with increased ventilation – 24 L/s per person compared to 12 L/s per person (131). Moreover, studies conducted in army barracks, jails, hospitals, and office buildings have shown that key building-related factors include ACH and the rate of air recirculation. Low ventilation and increased air recirculation enhance the potential for virus spread (132).

It is obvious that if you can remove, by ventilation or filtration, or inactivate the infectious particles in the air, you can reduce the risk of infection. On the other hand, ventilation may also result in transmission between rooms, e.g., in cruise ships and hospitals, e.g., (133). A recent systematic review concludes that viruses are inactivated by UV radiation (134). Naturally, potential adverse effects resulting from the use of UV radiation as a method of disinfection should also be carefully evaluated.

##### 3.3.1.2 Temperature and air humidity

*Temperature and air humidity* have been shown in several studies to affect both the survival of viruses and the recipient’s susceptibility to infection, e.g., (123). It seems, that viruses with lipid envelopes are more stable in low relative humidity (RH), whereas viruses without a lipid envelope are more stable in higher RH (135). Viruses with lipid envelope include influenza, RS- and coronaviruses, and they are therefore more stable in dry air (i.e., < 40% RH); whilst viruses such as rhinovirus and adenoviruses are more stable in humid air. Another general observation is that viruses typically demonstrate increased stability at lower ambient air temperatures (e.g., (136)), therefor virus present in aerosols may remain viable longer in cold air (although, high temperature increases the evaporation rate forming smaller aerosols). Conditions like this may appear in modern buildings conditioned with cold and dry air (24). New research shows that a large proportion of the droplets will quickly dry out and shrink into a small core (e.g., (122, 136)), which can remain floating for hours and spread over large distances indoors (i.e., available to be inhaled by and to infect other recipients). Here, temperature and air humidity are of great importance for how quickly the large droplets dry into small aerosols (19, 122, 136).

Thus, in view of the seasonal dependence of infection in the northern hemisphere with high incidence in the winter season [e.g., Wang et al. (19)], the positive effects of ventilation should be assessed against the negative effects of exposure to dry (cold) air. These negative effects are: 1) more stable virus (at least those possessing lipid envelope) and increased transmission and infectivity; 2) impaired airway functionality (mucociliary clearance and immune response) increasing the susceptibility of the virus recipient; 3) increased generation of virus droplets/aerosols in the airways of infected people; and, 4) faster evaporation of water to smaller aerosols, preventing sedimentation and increasing the floating time, as well as by high temperature (19, 122, 123, 136).

Raising indoor air humidity to counteract dry conditions could serve as a non-pharmaceutical approach to reduce the risk of infection. Maintaining relative humidity levels between 40 and 60% seems to be optimal for health, work performance, and minimizing the risk of infection (137). Studies implies that humidification may reduce absence from work. However, the epidemiological evidence of lower absenteeism is uncertain as concluded in a systematic review based on four out of the six controlled intervention studies (138). Evidence from experimental studies, however, is not included in this review. Elevation of the RH in preschool classrooms has elegantly demonstrated infection reduction in an intervention study by Reiman et al. (139). Regulating air humidity to around 45% RH during the cold season significantly decreased airborne influenza virus levels compared to control classrooms. Additionally, classrooms with increased humidity experienced lower pupil absenteeism.

Experimental studies revealed increased influenza virus transmission in low air humidity and low temperature conditions. Specifically, using a guinea pig model demonstrated highly efficient transmission at 5°C compared to 30°C. Dry conditions (20 and 35% RH) were more conducive to transmission than humid conditions (50% RH and 80% RH) (140).

#### 3.3.2 Preventive measures related to organization

##### 3.3.2.1 Social distance

*Social distance* is a continuum from complete isolation to close contact with many people (also called physical distance). The effectness of social distance to reduce the probability of spreading an infection depends on several factors. First, the nature of the infectious agent. As a large body of data suggests that the dominant route of transmission for most respiratory viruses is via large and small aerosols (103, 141–143), distance and barriers between the infected person and the recipient are obviously important. Secondly, as reviewed in the previous paragraph, environmental factors like ventilation, humidity and temperature influence the likelihood of viral transmission in the indoor setting (144, 145). A mathematical model involving physical distance and ventilation efficiency concludes that increased physical distance (e.g., halving occupant density) will result in a significantly reduced infection rate (20–40%) during the first 30 min (146).

Where aerosol-mediated transfer is the dominant route of transmission, the size and initial speed of the aerosols are of critical importance for their fate in the air, as well as the distance traveled by the aerosols and the change in size due to evaporation, which is influenced by ambient temperature and RH.

Hedin et al. found that more than 50 children in the DCC was of significant importance for sickness absence among the children (6), and in a study from United States the strongest predictor of illness risk was the number of other children in the room (147). A Danish study comprising about 900 children (< 3 years) in 24 different DCCs showed that children-age and the time they have been enrolled in the institution correlated with number of absence days due to illness. The overall illness was found to be 19.5 days per child per year. In addition, the size of the common area in the institution was of significance. For every increase of square meter per child the number of days absence due to illness was reduced with 10.8% (148).

##### 3.3.2.2 Exclusion/quarantine

A Japanese study compared an intervention group of employees who were asked to stay at home if household members had influenza-like-illness (ILI) symptoms during the 2009 to 2010 H1N1 influenza pandemic with a control group of employees who were not asked to stay at home. Employees were instructed to remain at home until 5 days after the household member(s) demonstrated resolution of symptoms or 2 days after the fever subsided. This quarantine/physical distancing intervention led to a reduction in influenza transmission to co-workers compared with workers in the control group. Yet, individuals who remained at home with their infected family members had a higher likelihood of contracting the infection (149).

Many countries have recommendations to exclude children from DDCs while they have respiratory symptoms (150). This makes sense, as respiratory infections are apparently most contagious in the first days after symptom onset. However, we have not identified studies that can quantify the evidence for this measure and exclusion of children with respiratory infections is only partly effective in reducing transmission as virus may be shredded several days before and after the presentation of symptoms, and many viruses can cause asymptomatic infections.

##### 3.3.2.3 Physical distance and larger and smaller aerosols

Many respiratory viruses, including SARS-CoV-2 and influenza virus, spread from an infected person to susceptible individuals via airborne aerosols. These aerosols span a wide range of sizes from smaller than 1 μm to 1 mm. Gravitational forces, drag forces, and evaporation control the transmission of respiratory aerosols. The larger aerosols will settle on surfaces (the fomites route of transmission) (151, 152).

As the risk of transmission of SARS-CoV-2, and likely most other respiratory-viruses, through fomites is low (106, 153), the focus will be on aerosol transmission. A model for direct transmission (larger aerosols) has been developed based on aerodynamics and including data on aerosol sizes, evaporation and viral load. The risk of infection was found to be influenced by both environmental conditions and the nature of respiratory activities, indicating the absence of a universally safe distance (154). Further, Ma et al. developed a model for direct transmission and “contacting distance” based on data from Wuhan beginning of year 2020 for the spread of SARS-CoV-19. Contact distance refers to “the extent to which people experience a sense of familiarity (nearness and intimacy) or unfamiliarity (fairness and difference) between themselves and people belonging to different social, ethnic, occupational and religious groups from their own.” According to the model, individuals should maintain a minimum distance of 1.7 meters. This is not a safe distance, but a distance that results in a basic reproductive number (R_0_) less than 1, meaning that the epidemic will die out (155). However, this model does not consider smaller aerosol transmission, which is highly important in indoor settings. Furthermore, high protein contents in the aerosols, important for the evaporation kinetics, have not been included in the modeling.

There is compelling evidence indicating that the predominant factor in the transmission of COVID-19, and likely numerous other respiratory viruses, is the indoor spread through small aerosols generated by speaking or breathing (20, 141, 156, 157). Respiratory aerosols, originating in the lungs and other parts of the respiratory systems, consist of ≥95% water at the time of their initial generation. The rate of aerosol dehydration depends, apart from the content of salts and proteins (25), on size, RH of the surrounding air and its temperature, but most aerosols will shrink to less than one third of initial size within a few seconds (122, 136).

Adherence to the “Six-Foot Rule” (recommendation from many authorities) would limit large-aerosol transmission but offer little protection of small-aerosol transmission. There was no significant difference in the number of COVID-19 cases among students or staff in districts that implemented a 3-foot minimum physical distancing policy compared to those with a 6-foot minimum distancing policy (158).

In summary, physical distancing decreases exposure from pathogens in small aerosols as well as in large particles, although small particles have a greater floating/spreading time.

#### 3.3.3 Preventive measures related to behavior

Behavior can have a major influence on the spread of infection. During the corona era, we have placed particular emphasis on hand hygiene, surface cleaning, and considerate coughing and sneezing etiquette, among various other measures. New knowledge has shown that respiratory activity, such as physical activity, talking and singing is important for the excretion of potentially infectious aerosols. The most important behavioral aspects in relation to DCCs are reviewed below.

##### 3.3.3.1 Hand hygiene

*Hand hygiene* has been known in the prevention of the spread of infectious diseases since the eighteenth century (159). However, there is large variation in the effect of hand hygiene. Hand hygiene may consist of washing with soap and water or using hand sanitizer or substances that are more aggressive in various formulations. Hand hygiene has been found to be more effective against gastrointestinal viruses and bacteria that infect via the fecal-oral route, than against respiratory viruses such as corona-, influenza-, and rhinovirus, whose primary route of transmission is airborne infection via aerosols (160–162).

Only a few studies have examined hand hygiene routines in community settings with emphasis on respiratory infections. A recent British study found that regular handwashing (6–10 times per day) was associated with significant lower risk of coronavirus infection, but no dose–response effect of handwashing was found (163). On the contrary, a study conducted in Sweden during the pandemic influenza season from September 2009 to May 2010 found no substantial decrease in acute respiratory infection rates among adults with an increased frequency of daily hand-washing (164).

In a recent systematic review, it was found that comparing hand hygiene intervention with a control group resulted in a 16% reduction in the number of individuals with respiratory infections in the hand hygiene group (settings comprised schools, childcare centers, homes, and offices) (165). However, when focusing on laboratory-confirmed influenza-like illness, the intervention showed little or no difference. Nevertheless, the aggregated data suggested that hand hygiene may reduce respiratory illness by 11%, though with high heterogeneity (165).

Another systematic review with focus on the effect of using hand sanitizer (rinse-free hand wash) among children found that rinse-free hand wash may reduce absenteeism caused by acute respiratory illness about 20% (166). Azor-Martinez et al. (167) successfully conducted an intervention study on children at the age of 0–3 years in DCCs in Spain. They documented a reduction of 23% in episodes of respiratory infections following a comprehensive intervention program comprising training of children, staff and parents using hand sanitizer (70% ethanol) 6–8 times a day over 8 months. The intervention group using soap and water had a non-significant reduction of 6%. In a study of Swedish children in DCCs Lennell et al. found a 12% reduction of absenteeism due to infections in the intervention group using hand sanitizer (70% ethanol) after regular hand washing compared to the group using only soap and water (168). The intervention ran over 30 weeks, included instructions, and monthly visits by a nurse to check that the instructions were followed. Disinfection of hands was estimated to be 2–6 times per day.

A similar study of pupils (age 5 to 15 years) in Danish schools was not able to demonstrate a difference between intervention group and control group in the same year. In this study, the intervention group was instructed to use hand sanitizer (87% ethanol) 3 times during school days and received instructions in proper use of hand disinfection (169). Also, a study conducted in Iceland failed to show a significant decrease in the incidence rates of illnesses associated with comprehensive hygiene intervention in DCCs over a period of 2.5 years. The interventions focused on both hand and environmental hygiene, among other elements, education, hand washing training, and the staff and preschool children were provided with and encouraged to use hand disinfectant (85% ethanol) in addition to hand wash. The authors state that the ineffective hygiene intervention in reduction of febrile, respiratory, or gastrointestinal illnesses, most likely was due to the “high standard of baseline hygiene practices at the DCCs” in Iceland (170). Likewise, a study in the Netherlands showed no evidence for an effect of the intervention, comprising education and high focus on hand hygiene, including hand ethanol-based sanitizer, on the incidence of episodes of diarrhea and common cold (171).

In a comprehensive Finnish intervention study, which encompassed various components such as hand washing, training in environmental hygiene, emphasis on ventilation, and isolation of children with symptoms of communicable diseases, a 26% reduction in absenteeism due to infections was observed among under 3-year-olds, but not among older children (172).

A randomized, controlled trial of children in child-care found that the ability of infection control techniques (training of child-care staff about transmission of infection, handwashing, and aseptic nose wiping) to reduce episodes of colds was limited to children 24 months of age and under (173). Furthermore, a comprehensive review established that “hygiene is particularly effective in DCCs with low standards of hygiene” (174).

Hence, to sum up the effect of hand hygiene as a non-pharmaceutical intervention for the prevention of respiratory viruses depends on several factors. Which virus we are focusing on, and the way the hand wash and/or sanitizer is carried out (duration and frequency). In addition, it is of great importance from which basic level the intervention starts, i.e., whether there is a high incidence of infectious diseases. Several studies suggest that interventions are more effective on younger children. Overall, it is not unrealistic that many DCCs will be able to achieve a reduction in the number of sick leave days among children and staff due to acute respiratory infections in the order of 10% by an ongoing hand hygiene program with good compliance.

##### 3.3.3.2 Hygiene practices (cleaning of toys etc)

Fomite mediated transmission can be an important pathway for some viral diseases. This route of transmission includes self-inoculation of viruses in the mouth, eye or nose after contact with contaminated surfaces. However, so far we lack convincing evidence of its significance for respiratory infections. Many studies have demonstrated isolation of viral RNA from surfaces for several days and some report viral viability in cell culture or other assays. Survival of the virus on surfaces may be influenced by environmental factors such as temperature, humidity, exposure to UV, and surface characteristics (175). A study revealed that SARS-CoV-2 experienced faster decay on nonporous surfaces when either humidity or temperature was elevated (176). Casanova et al. previously showed that surrogates for coronavirus (transmissible gastroenteritis virus (TGEV) and mouse hepatitis virus (MHV)) maintained viability for over a week on steel plates at 20 and 80% RH and 20°C. In contrast, at 50% RH, the viable virus decreased to less than 1% after 2 days (177).

A model of fomite transmission has been developed assuming that this transmission route contribute significantly. According to the model, in certain office settings, fomite transmission could be disrupted by hourly cleaning and disinfection, especially when coupled with reduced shedding. However, this approach would prove insufficient for DCCs and schools (178). This find is substantiated as an intervention study in 12 day-care nurseries in Denmark conclude that “Although cleaning and disinfection of toys every 2 weeks can decrease the microbial load in nurseries, it does not appear to reduce sickness absence among nursery children” (179). Finally, a recent systematic review concludes that the lack of evidence suggests that the risk of transmission of SARS-CoV-2 through fomites is low (106).

##### 3.3.3.3 Aerosol modifying activities

Various respiratory activities such as breathing, speaking, singing, coughing, sneezing, etc., contribute to the formation of aerosols with infectious content. The number of exhaled aerosols differs considerably between individuals and can vary from 20 particles per liter of exhaled air to several thousand (180). The particles are formed by inhalation and released by the subsequent exhalation and can carry viruses out of the lungs of infected persons (180). The deeper the expiration the larger the number of released particles during the following exhalation. Using a standardized breathing maneuver, Bake et al. observed significant inter-individual variation in the quantity of particles in exhaled air, averaging 10,000 particles/L. Additionally, the particle size distribution showed a slight shift towards larger particles with increasing age and lung size (181).

The number of aerosols generated during speech correlates with the loudness, ranging from 1 to 50 aerosols per second (182). These aerosols remain suspended in the air for extended periods of up to 9 h for SARS-CoV-2 (183) with a half-life of about 1 hour (184). The fate of the aerosols is determined by their size (i.e., generation and evaporation kinetics) and airflows until removed by ventilation or inhalation. Bazant and Bush have developed a guideline for indoor airborne transmission of SARS-CoV-2 based on mathematical modeling of viral spreading. Using the model, they infer that “the safe time after an infected individual enters a classroom, with 20 persons, is 1.2 h for natural ventilation and 7.2 h with mechanical ventilation” (assuming a “quiet classroom” with resting respiration). Further, they find that risk of infection increases linearly with the number of people in a room and duration of the presence. The overall take home message from their elaborate work is that, to reduce the risk of infection, it is advisable to avoid prolonged stays in densely populated areas. Rooms with ample volume and high ventilation rates are considered safer.

The likelihood of virus transmission is elevated when individuals have an elevated respiration rate, leading to increased pathogen output, as seen during activities such as exercise, singing, or shouting (156). These recommendations are probably valid for most respiratory viruses.

Data indicates that speaking and singing exhibit similar particle size distributions. Nonetheless, the loudness of vocalization can result in a 20–30 times increase in mass concentration of small aerosols, ranging from the quietest to the loudest volume. Breathing produces fewer and smaller particles than singing and speaking (35). Underpinned by the observation that engaging in karaoke (singing in the company of others, often in small rooms) involves an increased risk of spreading infections viruses (185). A study by Hersen et al. (186) showed that exhaled breaths from subjects with symptoms of respiratory infections contained more small aerosols (particles <1 μm) than exhaled breaths from healthy subjects.

##### 3.3.3.4 Physical exercise

Exercise can lead to a ventilation increase exceeding tenfold. A noteworthy study revealed a 132-fold increase in aerosol particle emission from rest to maximal exercise. This study was done on healthy subjects, and therefore we do not know the potential concentration of a pathogen in the exhaled aerosol particles and whether the risk of infection increases correspondingly with the number of aerosols (187).

##### 3.3.3.5 Toilet flushing

A recent systematic review concludes that toilet flushing can “result in widespread bacterial and/or viral contamination in washrooms” (188). Despite the potential for microbial aerosolization due to toilet flushing, no evidence of airborne transmission of respiratory pathogens was found in public restrooms. Similarly, Jones et al. review shedding of SARS-CoV-2 in feces and urine and its potential role in disease transmission. The conclusion is that even though fecal shedding of the virus can persist for several weeks, the likelihood of SARS-CoV-2 being transmitted via feces or urine appears much lower, in comparison to enteric viruses (e.g., norovirus) due to the lower relative amounts of virus present in feces/urine (189).

##### 3.3.3.6 Other measures

*Other measures* related to behavior that influence the risk of infection indoors may include practicing natural ventilation by opening windows and doors and spending as much time outdoors as possible (e.g., on the playground or on outings). In a Danish overview study, different evidence was found that children’s sickness absence was reduced by increased time outdoors and by fewer children per square meter. It was calculated that the number of sick days per child decreased by 10.8% for every square meter the group room area was increased per child (190). But there may be several factors that, in addition to infection with respiratory infections, affect these observations.

### 3.4 Seasonality

Seasonality describes variations in virus prevalence at regular intervals throughout the year. From a preventive perspective, knowledge of when a respiratory virus is expected to increase in prevalence will be of great value. In recent years, we have gained an increased knowledge of the mechanisms behind seasonal dependence. Three mechanisms have been proposed to elucidate the seasonality of respiratory viruses [reviewed in Moriyama et al. (123)]: (i) virus survival and transmissibility in relation to humidity and temperature; (ii) changes in human behavior (e.g., more indoor in winter, less air circulation, holiday gatherings etc.); and (iii) the impact of changing temperature and humidity on host defense mechanisms (i.e., airway functionality).

#### 3.4.1 Virus stability and transmissibility

Transmission of respiratory viruses occurs primarily through aerosols. The water content in these potentially infectious aerosols reduces due to evaporation once these aerosols have been exhaled into the surrounding air. This process is determined by the temperature and relative humidity of the surrounding. Larger aerosols deposit fast on surfaces due to gravity, whereas smaller aerosols have the capacity to persist in the air for hours and travel over more extensive distances.

Typically, viruses responsible for seasonal surges in the winter months in temperate regions exhibit greater stability and transmissibility in animal models under conditions of low temperature and humidity (191). Many studies have currently examined the influence of climate on the transmission and mortality rates of the ongoing SARS-CoV-2 pandemic, and the majority of them have found a correlation between increased SARS-CoV-2 transmission and low temperature and relative humidity (192, 193). Further, both SARS-CoV-2 and RV replicates more efficiently at the cooler temperature found in the upper respiratory tract (30, 87). For RSV it was found that every 5°C increase in temperature was linked to a reduced risk of 37%, although the mechanisms behind this observation have not been clarified (68); elevated temperature will decrease the viability, but at the same time increase the evaporation to smaller aerosols, which will float longer.

#### 3.4.2 Human behavior

The cold winter season in temperate climates can cause people to prioritize indoor activities, which can lead to crowding and increased virus transmission. In a large study of the adult urban population in seven European cities it was found, that on average people spend 90% of their time indoors (195). In general, the number of person-to-person contacts is greater on workdays than on holidays, suggesting that most viral transmissions occur on the job. However, travel and gatherings during holidays may lead to new contacts, thereby introducing infectious virus into new communities (196). The lower temperature may also give rise to reduced ventilation and opening of windows for reasons of heating costs resulting in increased infection risks. The use of HVAC systems, on the other hand, may introduce cold dry air during high temperature episodes, favoring the viability of enveloped viruses; likewise, ventilation with cold and dry outdoor air during winter will introduce heated air with low absolute humidity.

#### 3.4.3 Impact on host defense mechanisms

There is a growing focus on the influence of environmental conditions on the host’s antiviral defense mechanisms [reviewed by Moriyama et al. (123)].

The mucus lining in the upper airways acts as an initial barrier, trapping viruses before cell infection. Dry air hinders mucus flow, leading to delayed virus clearance, loss of cilia, and epithelial cell detachment, weakening the primary defense against viral infection in lower humidity conditions.

Cumulatively, the data suggest that low humidity and temperature play pivotal roles in the seasonality of epidemic human respiratory viruses. These conditions boost virus viability and transmission, create favorable indoor environments for viral spread, and hinder host cell immune responses.

## 4 Discussion and conclusion

The number of respiratory infections in a population will depend on the season, the circulating viruses, the environment and the preventive measures implemented locally and in the community. It seems that a handful of viruses account for most of the infections we experience in everyday life and in DCCs.

In Denmark and the other Nordic countries, children spend a large part of their time in DCCs, leading to a dramatic increase in the number of infections among children, especially during their first period. Moreover, employees working in DCCs experience high rates of absenteeism due to illness. The extensive financial and personal consequences of these infections affect not only the institutions and society but also the children and their parents.

Viral respiratory infections are the predominant cause of both children’s and employees’ illness. For many years, preventive measures have focused on hand and general hygiene as it has been the widespread perception that the primary route of transmission for the most common respiratory viruses is via hands and surfaces.

If you want to uncover a virus’ transmission pathway, you must design experiments that document this pathway and exclude others. Alternatively, one must be able to interrupt a given route of infection by a well-known mechanism. These scientific experiments are difficult to carry out in practice and many of the studies on which we base our knowledge go back many years, to a time when the detection and characterization of viruses was in its infancy.

There is no doubt that an infection can be induced by injecting live viruses into the nasal mucosa or through the eyes, but how realistic is this route if it requires fresh wet snot in large quantities? Modern techniques have supported the fact that many viruses remain infectious on non-porous surfaces for hours so the focus on good cleaning and hygiene must be considered an essential prevention measure.

There is also no doubt that many viruses can infect through the air. Early experiments with guinea pigs showed that the infection could be transmitted between cages several meters away. For viruses such as measles and rubella and the tuberculosis bacterium, it has been well known that the dominant route of transmission has been through airborne aerosols. The COVID-19 era has given us new knowledge, about not only the corona virus and its variants, but also concerning how these viruses are transmitted and which prevention measures are most efficient. Among other things, the virus’ ability of survival in the air and on surfaces, the excretion of small infectious aerosols by various activities such as breathing and talking, and the importance of temperature, humidity and targeted hygiene.

In particular, the airborne infection, which is difficult to control, has become increasingly important in the scientific literature. The focus has been on coughing and sneezing forming a large amount of large aerosols (droplets), which are excreted at high speed. While it was previously believed that large droplets would quickly settle and have limited range, recent research has shown that a substantial portion of these droplets can dry out and shrink into smaller aerosols, capable of remaining airborne for hours and spreading over significant distances indoors. Temperature and humidity play a vital role in the drying process and survival (decay).

Furthermore, activities like singing, speaking, and even normal breathing can release a considerable number of small aerosols that may contain infectious viruses. Notably, super-spreading events, where a few individuals infect a large number of people, have been linked to airborne transmission, as observed in previous outbreaks such as the SARS epidemic in 2003, MERS, Influenza, and the current COVID-19 pandemic (103, 197).

With the new knowledge and insight that a larger part of the infection of the most common respiratory viruses takes place via the air, one can approach prevention and reduction of infection more qualified. Improving air quality becomes a key preventive measure, achieved by reducing the occupant density or increasing air exchange through measures like opening windows, enhancing ventilation systems, or spending more time outdoors. Additionally, maintaining a specific level of humidity is important for the infectivity of viruses. Studies have shown that viruses with lipid membranes, including influenza-, corona-, and RS-virus, are most rapidly inactivated at a relative humidity (RH) of 40–60%.

However, it is important to note that most viruses can be transmitted before symptoms appear, and some infected individuals may remain asymptomatic throughout the course of the infection. Furthermore, evidence suggests that a small number of individuals contribute to the majority of infection spread, as observed in super-spreading events (198).

The duration of contact between an infectious individual and a susceptible recipient, as well as activities that generate aerosols, play a critical role in the likelihood of transmission. Understanding that loud singing and speaking significantly increase the number of potentially infectious aerosols in the air can help inform necessary precautions.

Increased knowledge of the seasonal patterns of respiratory viruses allows for more targeted prevention efforts. Understanding which viruses are most likely to be prevalent at a given time can guide the selection of appropriate disinfectants, ventilation strategies, or more comprehensive prevention measures. It has long been recognized that outbreaks of influenza, RS-virus, and human coronaviruses primarily occur during the winter season in temperate climates. Other respiratory viruses, such as adenoviruses and RVs, exhibit higher activity during spring or fall but can cause infections throughout the year depending on subtype and societal circumstances. For instance, the reopening of schools after COVID-19 lockdowns was found to be a significant risk factor for the rebound of RS-virus, and RV activity tends to increase when holidays end.

Furthermore, the ongoing COVID-19 pandemic has highlighted the constant emergence of new variants with different properties, particularly in RNA viruses. These changes can impact receptor binding and the affected areas of the respiratory tract. Additionally, they can influence the viral shedding from infected cells and the virus viability in the air or on surfaces.

The worldwide implementation of measures to control the spread of COVID-19 has proven effective, not only in reducing the transmission of SARS-CoV-2 but also in mitigating the spread of several other respiratory viruses. In Denmark, for example, the winter of 2020–2021 witnessed a significant decrease in influenza, RS-virus, and other respiratory infections. However, striking the right balance between preventive measures and the occurrence of respiratory diseases remains a challenge for societies. Understanding the transmission routes of respiratory viruses is crucial in making informed decisions and prioritizing prevention measures based on economic evaluation. Giving special attention to younger children, particularly in DCCs, may help reduce the overall burden on society. Studies have shown a particular increased rate of SARS-CoV-2 transmission among children aged 0 to 2 years at the beginning of winter, suggesting that preventive measures in this specific age group are of particular importance (199).

Until real-time monitoring of airborne viruses becomes technically and economically feasible (200), measuring indoor CO_2_ concentration can serve as a proxy for assessing the risk of indoor infection with respiratory viruses. Increased levels of CO_2_ indoors are typically associated with human exhalation, breathing, talking, and singing. An elevated CO_2_ level compared to outdoor levels can indicate a higher probability of inhaling breath exhaled by infected individuals, thereby increasing the risk of infection (201). By employing cost-effective and user-friendly CO_2_ monitors, it becomes feasible to evaluate the potential for airborne transmission in a room and implement preventive measures like enhancing ventilation, mask-wearing, or minimizing exposure time to infected individuals (202). In light of the recognition that the primary respiratory viruses spread through the air in small and large aerosols, recommendations from authorities in the future should place particular emphasis on indoor air quality and hygiene and with particular focus on the interplay between ventilation, temperature, and air humidity. An extensive study from Italy has demonstrated that effective ventilation leads to a reduction in the number of COVID-19 cases in schools (130). Further research is needed to examine the prevalence of respiratory infections in relation to various indoor ventilation qualities, with a focus on both CO_2_ concentration, particulate matter, and air humidity, in diverse climatic regions and societal contexts. Institutions such as day-cares, healthcare facilities, schools, and other social establishments where large numbers of individuals, particularly children, gather, are of particular importance in this regard.

In conclusion, understanding the transmission pathways and characteristics of respiratory viruses is crucial for implementing effective prevention and control measures. The focus should be on controlling the air quality reducing viral concentration and viability and ensure optimal airway functionality.

### 4.1 Limitations

This article adopts the format of a narrative review, considering the diverse and complex nature of exposures, respiratory tract infections, and potential preventive measures. Despite our efforts to carry out literature searches systematically, it is possible that we may have overlooked relevant studies. Moreover, our descriptions of respiratory viruses have primarily focused on the most prevalent ones, potentially neglecting other viruses that may have a significant impact on respiratory infections in specific geographical locations and time periods.

Additionally, our understanding has been influenced by emerging evidence suggesting that airborne transmission is likely the primary route for transmission of the most important respiratory viruses. This newfound knowledge may have inadvertently introduced bias into our descriptions.

## Author contributions

LA: Conceptualization, Funding acquisition, Writing – original draft, Writing – review & editing. KK: Writing – review & editing. LS: Writing – review & editing. KH: Writing – review & editing. BG: Writing – review & editing. PW: Writing – review & editing. AM: Conceptualization, Writing – review & editing.
